# Prevalence and risk factors of Rift Valley fever in humans and animals from Kabale district in Southwestern Uganda, 2016

**DOI:** 10.1371/journal.pntd.0006412

**Published:** 2018-05-03

**Authors:** Luke Nyakarahuka, Annabelle de St. Maurice, Lawrence Purpura, Elizabeth Ervin, Stephen Balinandi, Alex Tumusiime, Jackson Kyondo, Sophia Mulei, Patrick Tusiime, Julius Lutwama, John D. Klena, Shelley Brown, Barbara Knust, Pierre E. Rollin, Stuart T. Nichol, Trevor R. Shoemaker

**Affiliations:** 1 Uganda Virus Research Institute, Department of Arbovirology, Emerging and Re-emerging Infections, Entebbe, Uganda; 2 Centers for Disease Control and Prevention, Division of High Consequence Pathogens and Pathology, Viral Special Pathogens Branch, Atlanta, Georgia, United States of America; 3 University of California Los Angeles, Division of Pediatric Infectious Disease, Los Angeles, CA; 4 Centers for Disease Control and Prevention, Division of High Consequence Pathogens and Pathology, Viral Special Pathogens Branch, Entebbe, Uganda; 5 Kabale district Health Office, Kabale, Uganda; INDEPENDENT RESEARCHER, UNITED STATES

## Abstract

**Background:**

Rift Valley fever (RVF) is a zoonotic disease caused by Rift Valley fever virus (RVFV) found in Africa and the Middle East. Outbreaks can cause extensive morbidity and mortality in humans and livestock. Following the diagnosis of two acute human RVF cases in Kabale district, Uganda, we conducted a serosurvey to estimate RVFV seroprevalence in humans and livestock and to identify associated risk factors.

**Methods:**

Humans and animals at abattoirs and villages in Kabale district were sampled. Persons were interviewed about RVFV exposure risk factors. Human blood was tested for anti-RVFV IgM and IgG, and animal blood for anti-RVFV IgG.

**Principal findings:**

655 human and 1051 animal blood samples were collected. Anti-RVFV IgG was detected in 78 (12%) human samples; 3 human samples (0.5%) had detectable IgM only, and 7 (1%) had both IgM and IgG. Of the 10 IgM-positive persons, 2 samples were positive for RVFV by PCR, confirming recent infection. Odds of RVFV seropositivity were greater in participants who were butchers (odds ratio [OR] 5.1; 95% confidence interval [95% CI]: 1.7–15.1) and those who reported handling raw meat (OR 3.4; 95% CI 1.2–9.8). No persons under age 20 were RVFV seropositive. The overall animal seropositivity was 13%, with 27% of cattle, 7% of goats, and 4% of sheep seropositive.

In a multivariate logistic regression, cattle species (OR 9.1; 95% CI 4.1–20.5), adult age (OR 3.0; 95% CI 1.6–5.6), and female sex (OR 2.1; 95%CI 1.0–4.3) were significantly associated with animal seropositivity. Individual human seropositivity was significantly associated with animal seropositivity by subcounty after adjusting for sex, age, and occupation (p < 0.05).

**Conclusions:**

Although no RVF cases had been detected in Uganda from 1968 to March 2016, our study suggests that RVFV has been circulating undetected in both humans and animals living in and around Kabale district. RVFV seropositivity in humans was associated with occupation, suggesting that the primary mode of RVFV transmission to humans in Kabale district could be through contact with animal blood or body fluids.

## Author summary

Viral hemorrhagic fevers are known to cause high morbidity and mortality and pose a serious threat to human and animal populations in endemic countries. An outbreak of Rift Valley fever was detected in Kabale district in March, 2016 and identified the first human cases in Uganda since 1968. There was a need to perform a rapid assessment of the burden of Rift valley fever in Kabale district, identify undetected acute cases, identify risk factors associated with human disease, identify areas at high-risk or future infections, and to determine if this was a newly emerging infection or an endemic disease. Our study found the seroprevalence to be as high as 28% in humans and 36% in animals within some subcounties of Kabale district. Human seropositivity correlated with animal seropositivity, suggesting that animal to human transmission may be the predominant mode of virus spread. Our findings also suggest that this virus may have been endemic for many years prior to these human cases being identified.

## Introduction

Rift Valley fever virus (RVFV) is a single-stranded RNA virus in the order *Bunyavirales*, and recently classified in the *Phenuiviridae* family [1] and the *Phlebovirus* genus. RVFV causes disease in humans and animals [2], and is transmitted by mosquitoes to livestock such as sheep, goats, and cattle [3]. Competent mosquito vectors include species from the *Aedes*, *Culex*, *Anopheles*, *Eretmapodites*, *Mansonia*, and *Coquillettidia* genera [4, 5]. Humans can become infected with RVFV when they come into contact with blood or body fluids of infected livestock while caring for sick animals, assisting with animal birth, or slaughtering livestock; or through bites of infected mosquitoes. Occupations at the greatest risk of RVFV infection include herdsmen and butchers [6–8]. Raw milk or meat consumption are potential sources of RVFV, although transmission via these routes has not been confirmed.

In livestock, RVFV infection can cause increased abortions and stillbirths, and high mortality in neonates and juvenile animals. As a result, RVFV outbreaks can lead to significant economic losses [3, 9]. In humans, infection can range from asymptomatic or a mild flu-like illness to more severe disease that includes hepatitis, retinitis, or encephalitis [10]. Approximately 1% of human cases develop hemorrhagic disease, and an estimated 1–2% of cases are fatal. However, during an outbreak in Saudi Arabia, case fatality was as high as 14% [10].

There is no specific treatment for RVFV infection in humans or animals, but supportive care may prevent complications and decrease mortality [2]. Currently, no RVFV vaccine is approved for use in either humans or animals in North America or Europe [3]. Some inactivated and live-attenuated vaccines have been developed and have been efficacious in animals [11, 12]. Development of human RVFV vaccines has been challenging due to the safety of the vaccine. An experimental human vaccine, TSI-GSD200, has shown some utility in laboratory workers, but has not been used extensively in other settings [11]. To better understand the utility of RVFV vaccines in a particular setting, the prevalence of disease in humans and animals must first be understood.

Rift Valley fever (RVF) outbreaks were first reported in East Africa in the 1930s [13]. In 1997–1998, large RVF outbreaks in northeastern Kenya were associated with El Niño rains and floods, resulting in many deaths in livestock and humans [14, 15]. Infection in humans was highly associated with contact with livestock and animal body fluids. RVF outbreaks were not reported again in East Africa until 2006–2007, when large numbers of humans and livestock were infected in Kenya, Somalia, Tanzania, and Sudan [16, 17]. Studies following these outbreaks reported that being a herdsman and handling or consuming products from infected animals were major risk factors for human infection. Additionally, outbreaks in East African countries mainly occurred in areas with poor soil drainage and flat lowlands that are less than 500 m above sea level [18, 19]. Also, RVF outbreaks in humans and animals following flooding have occurred in Sudan [20], Saudi Arabia [21], Yemen (2000–2001), South Africa, and Egypt [22].

Uganda is an East African nation that shares borders with Kenya and Tanzania, and to date no large RVF outbreaks have been reported since 1968, when 7 human cases occurred near Entebbe [23]. However, a 2013 serological survey of goats in Ssembabule, Mpigi, Masaka, and Mubende districts in Uganda showed a seroprevalence of 9.8%, suggesting RVFV circulation [24].

The Uganda Virus Research Institute (UVRI) in Entebbe has been implementing laboratory-based surveillance for viral hemorrhagic fevers (VHF) in Uganda since 2010, which includes testing for RVFV [25]. In March 2016, UVRI confirmed 2 acute human RVF cases in Kabale district in the southwestern region of Uganda [26]. These were the first human RVF cases identified in Uganda since 1968 [23]. Because not all human or animal RVF cases are symptomatic, RVFV infections are often undetected. Thus, UVRI, the Ugandan Ministry of Health (MoH), Ugandan Ministry of Agriculture Animal Industry and Fisheries (MAIF), and the United States Centers for Disease Control and Prevention (CDC) collaborated on a study to assess the seroprevalence of RVFV in humans and animals living in and around Kabale district. The objectives of the study were to determine the sero-prevalence of RVFV in both humans and animals in Kabale and surrounding districts, identify risk factors and high-risk areas for RVFV, determine if RVF is emerging or endemic, and identify unrecognized RVF cases that may be related to the 2016 outbreak.

## Methods

### Ethics statement

Ethical approval for this study was granted from review by the UVRI Research Ethics Committee (UVRI REC: GC/127/16/03/551). Animal subjects work was conducted according to Uganda national guidelines and performed by officers from Kabale District and the Ministry of Agriculture, Animal Industries and Fisheries. The CDC National Center for Emerging and Zoonotic Infectious Diseases (NCEZID) Human Subjects office classified this project as non-research because the survey was a follow-up to the confirmed outbreak in Kabale district and the results of the study would assist the local health officials to target public health actions and interventions based on the serosurvey results (NCEZID: 032316TS).

### Study site and population

Kabale district is located in the southwestern corner of Uganda. According to the 2014 Uganda census, it has an estimated population of 534,160 people, with the majority living in a rural setting (457,592 of 534,160; 86%) [27]. The altitude of Kabale ranges from 1,219 m to 2,347 m above sea level. Agriculture is an important source of revenue in this region. Most families own goats, sheep, cattle, and pigs. In addition to agricultural lands, Kabale district also has areas with high-altitude rain forests and savannahs.

### Site selection strategy

**Fig 1:**
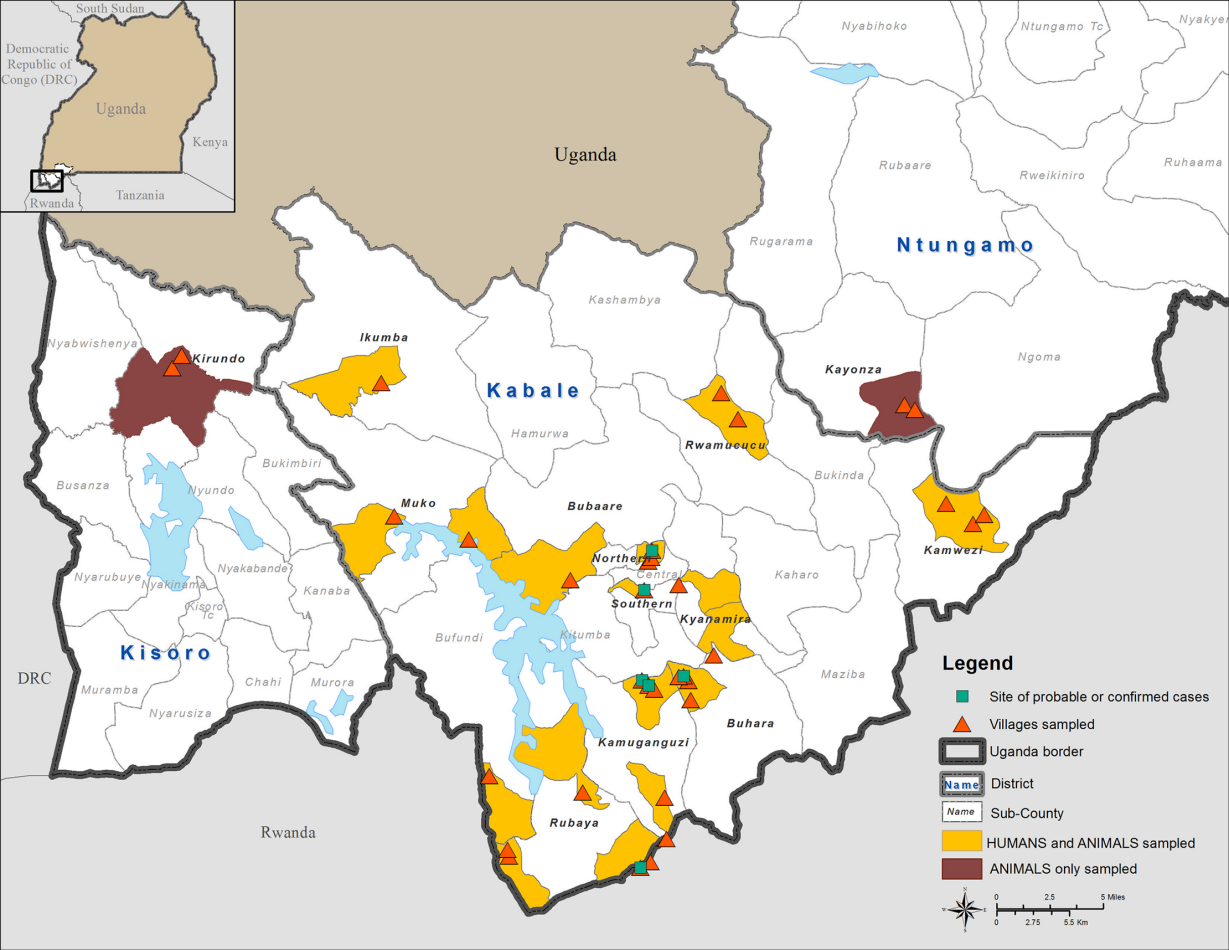
Map of Kabale district showing locations where either humans and animals, or animals only, were sampled in proximity to locations where confirmed acute or probable RVFV human outbreak cases were identified. This figure was created specifically for this manuscript in ArcGIS using open source data from ESRI and DIVA-GIS for the background layers, and GPS points collected in the field for the points. (ESRI—http://opendata.arcgis.com/about, DIVA-GIS—http://www.diva-gis.org/).

From April 1–12, 2016, 34 locations in and near Kabale district were selected for inclusion in the serosurvey by a multidisciplinary team consisting of individuals from Kabale district, Uganda MoH, Uganda MAIF, UVRI, and CDC (Fig 1).

Four categories of people and animals were targeted for sampling based on perceived risk of RVFV infection. These were: 1) Animal slaughter house (abattoir) workers and the animals (cattle, goats, and sheep) slaughtered at the abattoir; 2) persons and animals from villages that had confirmed or probable human RVF cases; 3) persons and animals from villages considered at-risk for RVF due to geographic conditions (see below); and 4) persons and animals from randomly selected villages with no reported RVF cases. Additionally, animals were sampled from neighboring districts of Ntungamo and Kisoro. Since several studies have demonstrated increased risk of RVFV exposure in butchers, we selected this as a high-risk group for sampling. The research team worked with Kabale district health and veterinary officials to select the study sites determined to be at risk for RVF; these sites were identified based on the terrain, propensity for flooding, human and animal population density, cooperation from the community, and sharing of international borders.

### Human participant recruitment strategy

When the team arrived at a selected location, local health workers assisted in recruiting participants for the serosurvey from the village by word of mouth. The questionnaire was written in English but was translated to the local language (Rukiga) and administered orally by local health workers (S1). Convenience sampling was used; all participants who presented to the sampling site were considered eligible for the study. All participation was voluntary and participants did not receive any compensation. Participants completed a consent form for the questionnaire, which was translated from English into local languages and explained by team members. Children older than 7 years were allowed to participate if a parent provided written consent. Blood samples were collected from all but 2 eligible participants identified.

### Animal sampling

Goats, sheep, and cattle that were brought to the collection site were sampled in relative proportion to the size of the herd at a given study site. Due to limited grazing areas in the Kabale region, few livestock owners maintain herds of more than 15 animals per species of cattle, sheep and goats. In cases where a livestock owner had less than 15 animals in a herd, all animals would be sampled. If an owner had more than 15 animals in a herd, only 25% of the herd was sampled. Only 3 of the households we sampled reported to have more than 15 animals per herd. We sampled all animals whose owners provided consent and presented their animals to the survey team. Both animals with a history of previous abortions or reported as having symptoms compatible with RVF, and apparently healthy animals of varied ages and sexes were sampled. Information collected about each herd included the general health of the herd, size of the herd, and grazing patterns. Because some humans and animals traveled from other subcounties to the location where the study was being administered, we did not collect both human and animal samples from all targeted subcounties.

### Blood collection and laboratory testing

One ~4 cc blood sample was collected from each human and animal participant for serological testing. Human specimens were tested by ELISA for anti-RVFV IgM and IgG, and animal specimens were tested for anti-RVFV IgG only. ELISA testing of both human and animal samples was performed at UVRI as previously described [28]. Human blood specimens that were IgM positive were subsequently tested by RT-PCR for RVFV-specific RNA targeting the L genome segment[29]. Briefly, following heat and detergent inactivation, specimens were tested by anti-RVFV-specific IgM and IgG ELISA using inactivated RVFV-infected Vero-E6 cell antigens, using 4 dilutions of each specimen (1/100, 1/400, 1/1600, and 1/6400). Titers and the cumulative sum optical densities of each dilution (SUMOD) minus the background absorbance of uninfected control antigen (adjusted SUMOD) were recorded. Samples were deemed positive if both the adjusted SUMOD and titer were above pre-established conservative cutoff values of ≥0.45 for IgM ELISA and ≥0.95 for IgG ELISA [28].

### Statistical analyses

Questionnaire data were entered into a Microsoft Excel worksheet and analyzed using Stata 13 (StataCorp LP, College Station, TX, USA). The significance of human risk factors associated with RVFV seropositivity was first determined using bivariate analysis based on Pearson’s χ^2^. A p-value <0.05 was considered statistically significant. After completing the bivariate analysis, the significance of independent variables as predictors was further assessed using multivariate logistic regression. All risk factors found to be significantly associated with human RVFV seropositivity were included in the multivariate logistic regression model. A Pearson’s χ^2^ goodness of fit test was done on the final model [30]. To determine if animal seropositivity at the subcounty level was significantly associated with human seropositivity within that subcounty, a multivariate logistic regression was performed adjusting for risk factors found to be significant in the bivariate analysis.

## Results

### Participant demographics

A total of 655 persons participated in the serosurvey (Table 1). Participants were recruited at the Kabale town abattoir (n = 117; 18% of participants), from villages where a recent acute RVFV case had been identified (n = 237; 37%), and from villages with no recorded outbreaks (n = 293; 45%). Most participants (n = 396; 60%) were aged 20–49 years and had completed primary education (n = 360; 55%). The most common occupation listed was farmer or herdsman (n = 335; 52%), and most individuals owned animals (60%). Contact with animals was common, with 78% (n = 508) of participants reporting contact with animals in the past year.

**Table 1:** Human demographics.

Variable	Number (%)
**Gender**	
Male Sex N (%)	426 (66%)
Female Sex N (%)	218 (34%)
**Mean age in years (inter-quartile range)**	40 years (27–50)
**Age group**	
7–19	65(10%)
20–49	396 (60%)
≥50	194 (30%)
**Education N (%)**	
None	154 (24%)
Primary	360 (55%)
Secondary and Post-Secondary	141 (21%)
**Marital Status N (%)**	
Single (including widowed and divorced)	167 (27%)
Married	441 (73%)
**Occupation N (%)**	
Herdsman/farmer	335 (51%)
Butcher	115 (18%)
Other	201 (31%)
**Own Domestic Animals**	
No Yes	264 (40%) 390 (60%)
**Contact with Animals**	
No	147 (22%)
Yes	508 (78%)

A total of 1,051 animals were sampled. Of these, 324 (31%) were cattle, 569 (54%) were goats, and 157 (15%) were sheep. Most were adults (n = 620; 59%) and a local breed (n = 829; 79%).

### Human and animal serology results

Of all persons tested, 13% (88/655) were RVFV seropositive. Three (0.5%) persons had anti-RVFV IgM only, 78 (12%) had IgG only, and 7 (1%) had both IgM and IgG. Two individuals positive for RVFV IgM also tested positive for RVFV RNA by RT-PCR, suggesting active infection at the time of sampling. The 3 IgM-only positive individuals (a trader, a housewife, and a farmer) were all from the village in which one of the initial acute human RVFV cases was living but were not related to the initial acute RVF case. No persons under 20 years of age were RVFV seropositive, while 17% (n = 66) of individuals aged 20–49 years were seropositive (Table 2). Of individuals 50 years and older, 11% (n = 22) were seropositive. Butchers were the most likely to be RVFV seropositive, with 35% showing evidence of seropositivity. Other occupations evaluated for RVFV seropositive include farming at 10%, housewife at 8% (4/49), teacher at 18% (2/11) and trader at 12.5% (3/24). In the bivariate analysis, older age groups (χ^2^ = 14.4; p = 0.001), male sex (χ^2^ = 11.9; p = 0.001), occupation as a butcher (χ^2^ = 54.7; p < 0.001), history of slaughtering or butchering animals (χ^2^ = 23; p < 0.001), and preparing raw meat (χ^2^ = 13; p < 0.001) were all significiently associated with an increased risk of RVFV seropositivity (S1 Table).

**Table 2:** RVF seropositivity of human participants.

	Seronegative	Seropositive	IgM +	IgG +	IgM+ IgG +
**Sex**					
Male	355 (83%)	71 (17%)	0	67 (16%)	3 (1%)
Female	203 (93%)	15 (7%)	3 (1%)	9 (4%)	4 (1%)
**Age Group**					
Age 7–19	65 (100%)	0	0	0	0
Age 20–49	330 (83%)	66 (17%)	3 (1%)	57 (14%)	6 (2%)
Age ≥50	172 (89%)	22 (11%)	0	21 (11%)	1 (1%)
**Occupation**					
Herdsman/Farmer Butcher	302 (90%) 75 (65%)	33 (10%) 40 (35%)	1 (0.3%) 0	30 (9%) 38 (33%)	2 (0.6%) 2 (2%)
Other*	186 (93%)	15 (7%)	2 (1%)	10 (5%)	3 (1.5%)
**Own Animals**					
No	236 (89%)	28 (11%)	1 (<1%)	25 (9%)	2 (1%)
Yes	330 (85%)	60 (15%)	2 (1%)	53 (14%)	5 (1%)
**Contact with Animals**					
No	129 (88%)	18 (12%)	2 (1%)	14 (10%)	2 (1%)
Yes	438 (86%)	70 (14%)	1 (<1%)	64 (13%)	5 (1%)

* (house wife, trader, teacher, student, Carpenter, casual laborer, driver, mason and health workers)

In the multivariate logistic regression, being a butcher and handling raw meat were significantly associated with RVFV seropositivity, with an adjusted OR of 5.1 (95% CI 1.7–15.1; p = 0.003) and 3.4 (95% CI 1.2–9.8; p = 0.024), respectively (Table 3). Age, sex, slaughtering/butchering, and contact with animals through grazing were not significantly associated with RVFV seropositivity in the multivariate model.

**Table 3:** Multivariate analysis of risk factors for RVF seropositivity in humans.

Variable	Seronegative	Seropositive	Adjusted Odds Ratio (OR)*	p-value	95% (CI)
**Sex**					
Female	203 (93%)	15 (7%)	Ref		
Male	355 (83%)	71 (17%)	2.3	0.073	0.92–5.7
**Age (as a continuous variable)**				0.054	
**Occupation**					
Other	186 (93%)	15 (7%)	Ref		
Herdsman/Farmer	302 (90%)	33 (10%)	1.1	0.78	0.47–2.7
Butcher	75 (65%)	40 (35%)	5.1	**0.003**	**1.7–15.1**
**Live Animal Contact**					
Grazing	319 (91%)	33 (9%)	0.84	0.62	0.41–1.7
**Meat Preparation**					
Slaughtering/butchering	185 (78%)	53 (22%)	0.67	0.40	0.27–1.7
Handling raw meat	362 (84%)	71 (16%)	3.4	**0.024**	**1.2–9.8**

*Adjusting for age as a continuous variable in addition to the variables listed in the table

Of all animals tested, 13% (133/1051) were RVFV seropositive. Seropositivity varied by species, sex, and age group among animals both in a bivariate analysis and the multivariate logistic regression (S2 Table; Table 4). Cattle showed significantly higher odds of being seropositive even after adjusting for age and sex (OR 9.1; 95% CI 4.1–20.5; p < 0.001). Adult animals and females also had significantly higher odds of being RVFV seropositive, with OR 3.0 (95% CI 1.6–5.6; p = 0.001) and OR 2.1 (95% CI 1.0–4.3; p = 0.04), respectively.

**Table 4:** Multivariate logistic regression model for RVFV seropositivity in livestock.

Variable	Seronegative	Seropositive	Adjusted OR*	95% CI	P-Value
**Species**					
Sheep	151 (96%)	7 (4%)	Ref		
Goat	529 (93%)	40 (7%)	1.9	0.87–4.6	0.1
Cow	238 (73%)	86 (27%)	**9.1**	**4.1–20.5**	**<0.001**
**Age**					
Infant	215 (94%)	13 (6%)	Ref		
Middle	188 (93%)	14 (7%)	1.0	0.47–2.3	0.92
Adult	514 (83%)	106 (17%)	**3.0**	**1.6–5.6**	**0.001**
**Sex**					
Male	173 (94%)	10 (5%)	Ref		
Female	721 (86%)	121 (14%)	**2.1**	**1.0–4.3**	**0.04**

*Adjusted for species, age and sex

**Fig 2:**
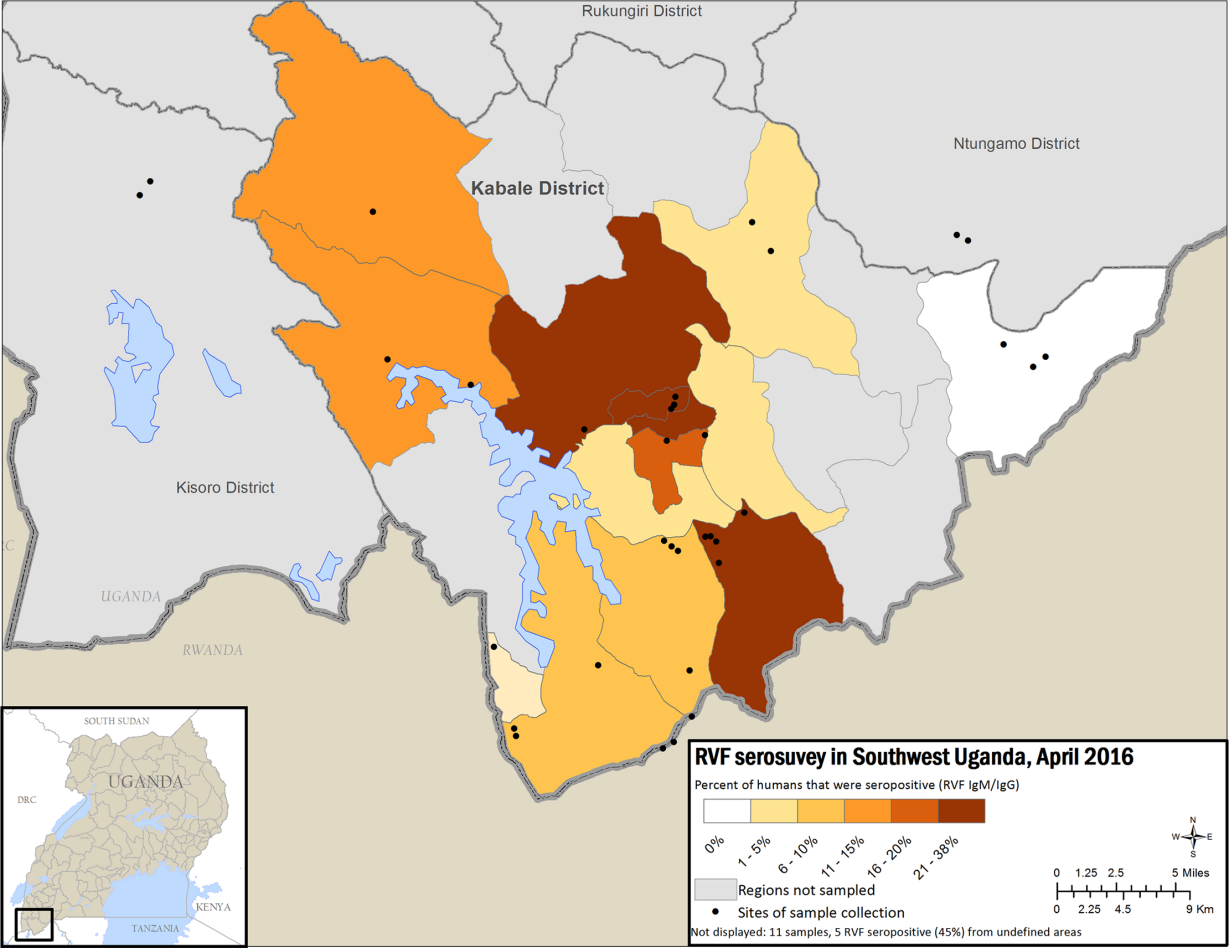
Map of Kabale district showing percent RVFV seropositivity for human samples by sub-county with less seropositivity in lighter colors and increasing seropositivity in darker color. This figure was created specifically for this manuscript in ArcGIS using open source data from ESRI and DIVA-GIS for the background layers, and GPS points collected in the field for the points. (ESRI—http://opendata.arcgis.com/about, DIVA-GIS—http://www.diva-gis.org/).

**Fig 3:**
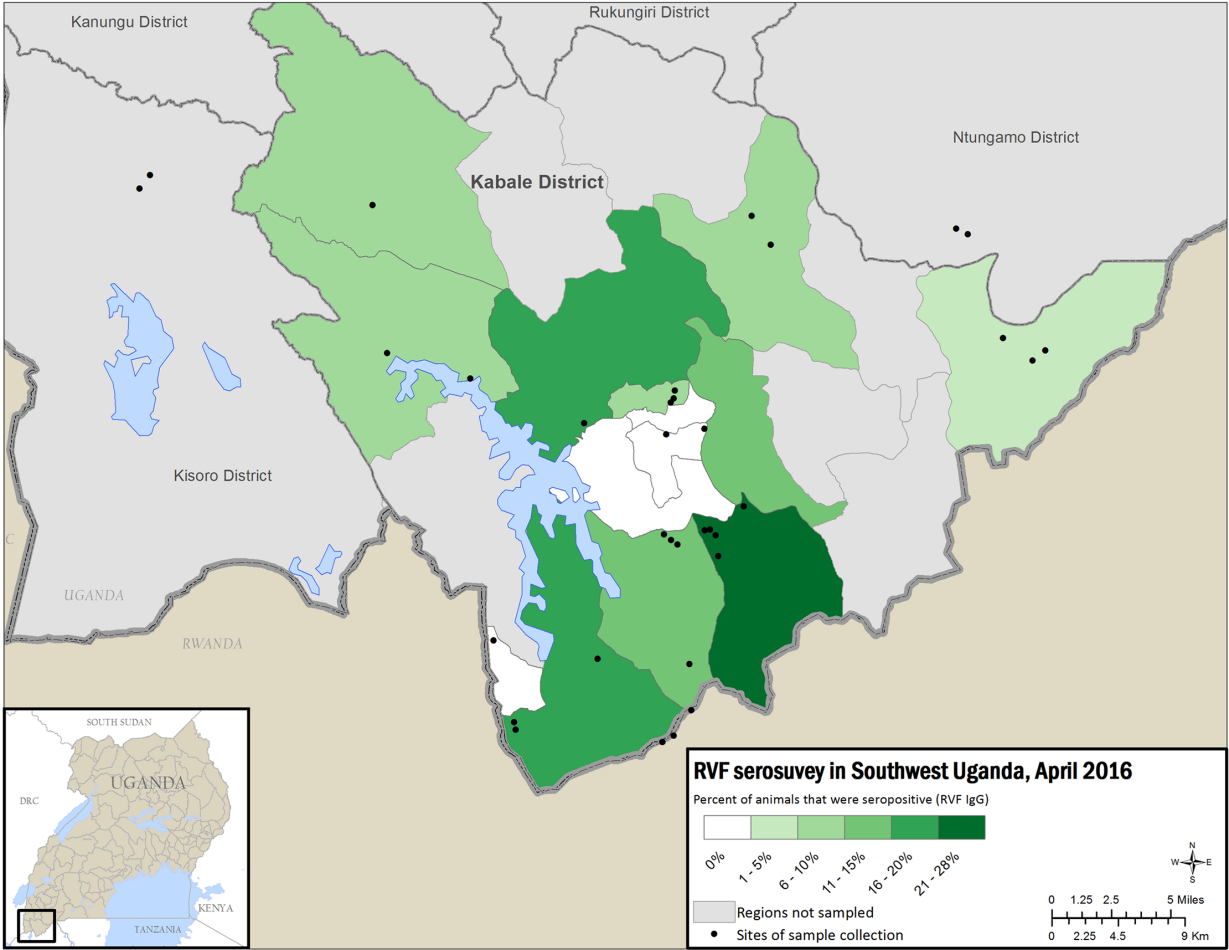
Map of Kabale district showing percent RVFV seropositivity for animal samples by sub-county with less seropositivity in lighter colors and increasing seropositivity in darker color. (ESRI—http://opendata.arcgis.com/about, DIVA-GIS—http://www.diva-gis.org/).

Human and animal seropositivity varied by subcounty (Figs 2 and 3), ranging 0–36% (standard deviation 11; mean 14%), whereas animal seropositivity ranged 4–28% (standard deviation 7; mean 12%) (S3 Table). Human RVFV seropositivity was significantly associated with close contact with cattle (P-value = 0.003), but shown not to be significant for contact with small ruminants (p-value = 0.06) (S1 Table). The sub-counties with the highest seroprevalence included the Kabale town council, where the main abattoir is located, and subcounties near bodies of water or wetlands.

### Human and animal serology association

The association between animal seropositivity and human seropositivity within a subcounty was examined using multivariate logistic regression, adjusting for contact with raw meat and occupation, because these were found to be significant risk factors in the univariate analysis. Human seropositivity within a subcounty was found to be associated with animal seropositivity, with OR of 1.1 (95% CI 1.0–1.1; p < 0.001).

## Discussion

Although RVFV had not been detected in Uganda since 1968, our study demonstrates that it has likely been enzootic in Kabale district for some time. Overall, we found evidence of RVFV seropositivity in 13% of humans and animals sampled. Our study also showed that butchers and those who handled raw meat were most likely to be RVFV seropositive.

Similar risk factors for RVFV seropositivity have been reported in previous studies. In a 2015 study in Kenya, LaBeaud and colleagues found that male sex, increased age, history of slaughtering livestock, history of malaise, and poor measured visual acuity were all factors for increased seropositivity [8, 31]. Although we did not find an association with sex and RVFV seropositivity after adjusting for other factors, such as occupation, we did find an association with being a butcher (i.e., someone who cuts meat either at home or at a slaughterhouse) and RVFV seropositivity. Previous studies also found that drinking raw milk may be associated with RVFV seropositivity [32, 33], but we were not able to find this association, likely because few individuals (36; 5%) reported drinking raw milk. Anecdotally, individuals reported not drinking raw milk due to concerns about *Brucellosis* infection. We did not find a significant association between age and seropositivity, but interestingly, no persons younger than 20 years were found to have evidence of RVFV infection. This may be because only 6 individuals below the age of 20 reported having close contact with livestock with the majority in this age group reporting to be attending school, greatly reducing their risk of exposure to potentially infected livestock.

Figs 2 and 3 show high seroprevalence, both in humans and animals, in 2 subcounties: Buhara subcounty, bordering Rwanda, and Bubare subcounty, near Kabale town. Kabale town contains the main abattoir, a likely source of RVFV infection in humans. However, Buhara and Bubare subcounties are connected by the primary North-South highway between Kabale town and the Rwandan border, a transportation corridor that could have served as a possible source of introduction of RVFV into Kabale district through livestock trade. A serosurvey of livestock conducted in Rwanda showed overall seropositivity of 16.8%, with districts closest to Tanzania showing the highest seropositivity and the 2 districts closest to the Ugandan border having the lowest seropositivity [34]. In addition, evidence of previous RVFV circulation and infection of livestock from samples collected in 2009 in goats from the Southeastern districts of Ssembabule, Mpigi, Masaka and Mubende shows a total seroprevalence of approx. 10% [24]. Although the testing methodologies employed for these samples was different than the one employed in our study, evidence of seropositivity in livestock may begin at earliest in 2009, or possibly earlier, and may suggest that RVFV was introduced into this region following outbreaks in neighboring countries and maintained through low level inter-epidemic transmission [34]. However, because there are no published reports of RVF seropositivity prior to 2009, we cannot be certain there was no widespread circulation of RVFV before that time. Our study suggests that RVFV transmission to humans in Kabale district is primarily due to exposure to the blood and body fluids of infected livestock, given that butchers and those handling raw meat were most likely to be RVFV-seropositive. Additionally, we found that human seropositivity was significantly associated with livestock seropositivity in each subcounty. Cattle density has also been previously associated with RVF seropositivity in RVFV models [35]. In our study, significantly more cattle were seropositive (27%) than goats (7%) or sheep (4%). This difference could be due to mosquito feeding behavior, as mosquitoes tend to select large and ornamented species [36]. Also, cattle are kept longer compared to goats and sheep hence are available to exposure to mosquito bites increasing their chances of being seropositive, however other factors such as mosquitoes species involved could be playing a role in Kabale district. For example, mosquitoes may have a preference for biting cattle compared to other ruminants. Furthermore, sheep and goats are usually kept indoors, especially at night, while cattle are rarely sheltered in Kabale district and thus exposed to nighttime-biting mosquitoes. Mosquitoes collected after outbreak investigations were mostly animal-specific feeders rather than human-specific (Julius Lutwama, personal communication), indicating that RVFV is primarily transmitted by mosquitoes within animal and human populations, however, humans are also infected from direct contact with infected animals. The mosquito species that were trapped in this region during the 2016 RVF outbreak include mainly *Aedes* and *Culex* species. Further investigations of the relative contribution of mosquitoes and livestock in RVFV transmission are needed.

The high seroprevalence in livestock seen in Kabale town is likely due to the main abattoir, where a majority of the animals in our survey were sampled. Animals, primarily from the neighboring subcounties, but also from more distant subcounties, are brought there for sale and slaughter. The high seroprevalence in animals can be attributed to the convergence of these animals from throughout the district in one location. This high seroprevalence in abattoir animals may be related to beef production dynamics in Uganda where older animals that are no longer of reproduction value are sold off by farmers for slaughter, hence more likely to be seropositive as stated above. The corresponding high seroprevalence seen in the abattoir workers can also be attributed to this, as well as daily occupational exposure to infected animal blood and body fluids.

Comparing our results with serological studies conducted in the neighboring countries of Kenya and Tanzania, where RVFV is endemic, provides some insight into our human serosurvey findings. In a study in coastal area of Kenya from 2009–2011 [37], RVFV seroprevalence in humans was lower (1.8%) than in our study. Similarly, the seroprevalence in humans was only 5.2% in Mbeya region in Tanzania [38], compared to 13% in our study. We think this is mainly because we collected samples after a confirmed outbreak or uptick in inter-epidemic transmission, unlike in the Kenya study. However, seroprevalence was higher in domestic ruminants in another study in Garissa, Kenya (27.6%) [39] than in our study (13%). Generally, seroprevalence in both animals and humans is expected to be higher in RVFV-endemic regions of Kenya and Tanzania than what we found in this study, but the risk factors identified were the same–mainly, contact with livestock. In a study in nearby Rwanda, the seroprevalence in cattle was 16.8%, slightly lower than 27% in our study [34]. However, these differences could be attributed to various factors, including the serological testing protocol used, which may differ in specificity and sensitivity. Additionally, our study identified local variations in RVFV seroprevalence, so the sampling strategy may also affect seropositivity results.

The extent of RVFV infection in Kabale district was greater than anticipated (as high as 36% in Bubare subcounty, and 28% in Buhara subcounty in animals) considering that the acutely identified human cases were the first to be laboratory confirmed in Uganda since 1968. The presence and percentage of RVFV-specific IgM, IgG and PCR-positive samples in persons in Kabale district indicates emergence of RVFV in Kabale is localized and that specific geographical locations may show varying levels of transmission or exposure to recently infected livestock. The level of IgG seropositivity indicates that RVFV has been circulating in the district for some time and further studies are needed to identify when this introduction may have occurred.

Our study had some limitations. Prior to initiating the survey, no reliable estimates of animal and human RVFV seropositivity existed, so we do not know how representative our samples are of the general population in Kabale district. In analyzing the correlation between human seropositivity and animal seropositivity by subcounty, we assume that seropositive humans were most likely to be exposed to RVFV within the subcounty of their residence, and did not take into account possible exposure in other locations.

Uganda has had a formal dedicated VHF surveillance program coordinated by UVRI and the MoH since 2011. During the past several years, 11 independent VHF outbreaks have been detected and confirmed by the UVRI VHF laboratory, including an outbreak of Marburg virus in 2012 that was first detected from a case in Kabale district [40]. Because of the previous experience and enhanced training and awareness, surveillance for VHF cases has become a priority, and healthcare workers were able to quickly identify the initial RVFV cases and immediately send samples for laboratory confirmation. Enhanced VHF and RVFV surveillance and health education must be continued given the potential for future RVFV re-emergence. Alongside this serosurvey, a knowledge, attitude practice (KAP) study was conducted in Kabale district and the findings of this KAP study has been used to design health education materials targeting different stakeholders [41]. For example, the education materials targeting farmers and butchers emphasize reporting of any sick animals to veterinarians, washing hands after touching raw meat or milk, cooking meat and milk thoroughly, using of mosquito bed nets and wearing of more protective clothing when working in high risk areas.

WHO recommends continued surveillance of RVFV during both outbreak and inter-epidemic periods [42]. Although RVFV rarely causes death in infected persons, the economic consequences due to loss of livestock and animal abortions can be devastating in agricultural communities [43]. Further studies are needed to fully understand the enzootic and endemic presence of RVFV in Kabale and surrounding districts. A longitudinal study is underway in Uganda to obtain samples from animals in Kabale district and throughout Uganda over several years to determine the endemicity and spread of RVFV over time. This will help determine the regions are at risk for RVFV outbreaks, so resources and surveillance efforts can be targeted to detect emergent cases and initiate any necessary control efforts.

## Supporting information

S1 Checklist STROBE items that should be included in reports of cross-sectional studies. (DOCX)

S1 Table Bivariate analysis of RVF seropositivity risk factors in humans. (DOCX)

S2 Table Bivariate analysis for risk factors for RVF seropositivity in animals. (DOCX)

S3 Table Human and animal seropositivity by subcounty. (DOCX)
