# Supplementary material for: Prevalence and risk factors of Rift Valley fever in humans and animals from Kabale district in Southwestern Uganda, 2016
Source: PLoS Negl Trop Dis. 2018 May 3;12(5):e0006412. doi: 10.1371/journal.pntd.0006412 (PMC5953497; doi:10.1371/journal.pntd.0006412)
Supplement: S1 Checklist — (DOCX) [file pntd.0006412.s001.docx]

S1 Checklist: STROBE Items that should be included in reports of ***cross-sectional studies***

|  | Item No | Recommendation |
| --- | --- | --- |
| **Title and abstract** | 1 | (*a*) Abstract, paragraph 1 |
|  |  | (*b*) Abstract, paragraph 2-4 |
| Introduction | | |
| Background/rationale | 2 | Introduction, paragraph 1-5 |
| Objectives | 3 | Introduction, paragraph 6 |
| Methods | | |
| Study design | 4 | Methods, paragraph 2 |
| Setting | 5 | Methods, paragraph 2 |
| Participants | 6 | Methods, paragraph 3-5 |
| Variables | 7 | Methods, paragraph 7 |
| Data sources/ measurement | 8* | Methods, paragraph 7 |
| Bias | 9 | Methods, paragraph 7 |
| Study size | 10 | Methods, paragraph 3-5 |
| Quantitative variables | 11 | Methods, paragraph 7 |
| Statistical methods | 12 | Methods, paragraph 7 |
|  |  | Methods, paragraph 7 |
|  |  | Methods, paragraph 7 |
|  |  | Methods, paragraph 7 |
|  |  | Methods, paragraph 7 |
| Results | | |
| Participants | 13* | Results, paragraph 1 |
|  |  |  |
|  |  |  |
| Descriptive data | 14* | Results, paragraph 1 |
|  |  |  |
| Outcome data | 15* | Results, paragraph 1 |
| Main results | 16 | Results, paragraph 2-3 |
|  |  |  |
|  |  |  |
| Other analyses | 17 | Results, paragraph 4 |
| Discussion | | |
| Key results | 18 | Discussion, paragraph 1 |
| Limitations | 19 | Discussion, paragraph 8 |
| Interpretation | 20 | Discussion, paragraph 1 |
| Generalisability | 21 | Discussion, paragraph 8 |
| Other information | | |
| Funding | 22 | Funding, paragraph, 1 |
