# Supplementary material for: Prevalence and risk factors of Rift Valley fever in humans and animals from Kabale district in Southwestern Uganda, 2016
Source: PLoS Negl Trop Dis. 2018 May 3;12(5):e0006412. doi: 10.1371/journal.pntd.0006412 (PMC5953497; doi:10.1371/journal.pntd.0006412)
Supplement: S1 Table — (DOCX) [file pntd.0006412.s002.docx]

S1 Table. Bivariate analysis of RVF Seropositivity Risk Factors in Humans

|  | **Seronegative** | **Seropositive** | **X^2^** | **p-value** |
| --- | --- | --- | --- | --- |
| **Sex**  Male  Female | 355 (83%)  203 (93%) | 71 (17%)  15 (7%) | **11.9** | **0.001** |
| **Age Group**  Age 7-19  Age 20-49  Age ≥50 | 65 (100%)  330 (83%)  172 (89%) | 0  66 (17%)  22 (11%) | **14.4** | **0.001** |
| **Occupation**  Herdsman/Farmer  Butcher  Other | 302 (90%)  75 (65%)  186 (93%) | 33 (10%)  40 (35%)  15 (7%) | **54.7** | **<0.001** |
| **Own Animals**  No  Yes | 236 (89%)  330 (85%) | 28 (11%)  60 (15%) | 3.1 | 0.079 |
| **Contact with Animals**  No  Yes | 129 (88%)  438 (86%) | 18 (12%)  70 (14%) | 0.23 | 0.63 |
| **Type of Live Animal Contact**  Milking  Grazing  Grooming  Caring for sick  Birth | 64 (89%)  319 (91%)  117 (89%)  97 (86%)  181 (86%) | 8 (11%)  33 (9%)  14 (11%)  16 (14%)  30 (14%) | 0.54  **20**  1.5  0.0089  0.26 | 0.46  **<0.001**  0.22  0.93  0.61 |
| **Meat Preparation**  Slaughtering/butchering  Handling raw meat | 185 (78%)  362 (84%) | 53 (22%)  71 (16%) | **23**  **13** | **<0.001**  **<0.001** |
| **Food/milk consumption**  Raw milk consumption  Raw meat consumption | 32 (89%)  25 (83%) | 4 (11%)  5 (17%) | 0.18  0.27 | 0.67  0.6 |
