# Supplementary material for: Prevalence and risk factors of Rift Valley fever in humans and animals from Kabale district in Southwestern Uganda, 2016
Source: PLoS Negl Trop Dis. 2018 May 3;12(5):e0006412. doi: 10.1371/journal.pntd.0006412 (PMC5953497; doi:10.1371/journal.pntd.0006412)
Supplement: S2 Table — (DOCX) [file pntd.0006412.s003.docx]

S2 Table. Bivariate Analysis for Risk Factors for RVF Seropositivity in Animals

|  | **Seronegative** | **Seropositive** | **X^2^** | **P-Value** |
| --- | --- | --- | --- | --- |
| **Species**  Sheep  Goat  Cow | 151 (96%)  529 (93%)  238 (73%) | 7 (4%)  40 (7%)  86 (27%) | **82** | **<0.001** |
| **Age**  Infant  Middle  Adult | 215 (94%)  188 (93%)  514 (83%) | 13 (6%)  14 (7%)  106 (17%) | **27** | **<0.001** |
| **Gender**  Male  Female | 173 (94%)  721 (86%) | 10 (5%)  121 (14%) | **10.7** | **0.001** |
