# Supplementary material for: Prevalence and risk factors of Rift Valley fever in humans and animals from Kabale district in Southwestern Uganda, 2016
Source: PLoS Negl Trop Dis. 2018 May 3;12(5):e0006412. doi: 10.1371/journal.pntd.0006412 (PMC5953497; doi:10.1371/journal.pntd.0006412)
Supplement: S3 Table — (DOCX) [file pntd.0006412.s004.docx]

S3 Table. Human and Animal Seropositivity by SubCounty

| **Subcounty** | **Number of Animals Sampled** | **Animal Seropositivity**  **N(%)** | **Number of Humans Sampled** | **Human Seropositivity**  **N(%)** |
| --- | --- | --- | --- | --- |
| Bubaare | 22 | 4 (18%) | 36 | 13 (36%) |
| Buhara | 86 | 24 (28%) | 76 | 17 (22%) |
| Ikumba | 87 | 7 (8%) | 49 | 7 (14%) |
| Kabale Northern | 26 | 2 (8%) | 61 | 17 (28%) |
| Kamuganguzi | 128 | 17 (13%) | 53 | 5 (9%) |
| Kamwezi | 77 | 3 (4%) | 49 | 0 |
| Kyanamira | 128 | 15 (12%) | 49 | 2 (4%) |
| Muko | 69 | 7 (10%) | 51 | 7 (14%) |
| Rubaya | 157 | 26 (16%) | 73 | 5 (7%) |
| Rwamacucu | 81 | 6 (7%) | 50 | 1 (2%) |
